# The Indicated Remedy in Diseases of Women*Read before the Kansas State Homœopathic Society, May, 1895.

**Published:** 1895-08

**Authors:** Frances M. W. Jackson

**Affiliations:** Emporia, Kansas


### THE INDICATED REMEDY IN DISEASES OF WOMEN.*

FRANCES M. W. JACKSON, M. D., EMPORIA, KANSAS.

The title of this paper ought to sound strange to us. If we practice Homœopathy what should we do but give the indicated remedy, in this as in every other class of cases? And yet, judging from the general tone of our current homœopathic literature, particularly from sources which claim this class of diseases as a specialty, one would almost think that the indicated remedy was the last thing to be thought of, if thought of at all.

I read long articles on various branches of this specialty, many of them giving most minute and explicit directions as to certain surgical measures, or topical applications, or electrical treatment, and closing up generally with a paragraph something like this: "A few of the most common remedies ordinarily found useful are"—and here follows the mention of a few remedies, sometimes with brief indications for their use, often their names only. In either case the impression is given that the mechanical and topical measures are of paramount importance, and that internal treatment is secondary and of somewhat doubtful value.

What wonder that a physician should lack confidence in the power of the indicated remedy if he has already administered to his patient, through the delicate and quickly absorbing mucous surfaces of the reproductive tract a half-dozen or more

---

* Read before the Kansas State Homœopathic Society, May, 1895.

powerful and crude drugs, neither one of which has been given with reference to its similarity to the case?

Under such circumstances, and with such rude interference with the processes of nature, there is no opportunity of a fair test of the indicated remedy, although it is truly surprising how lenient and forgiving nature often is under such abuse. In spite of all such hindrances, the administration of the indicated remedy, after other measures have proved futile and been discontinued, is often attended with prompt and successful results.

Since this is true, as is demonstrated daily in the practice of many physicians, how does it happen that such practices as are detailed in our homœopathic journals should ever have been introduced? Surely they are no part of the system which Hahnemann gave to us. The title-page of the journal and the usual mention of a few homœopathic remedies at the close of the article are often the only distinguishing characteristics, and the only indices of the particular school of practice to which the writer claims to belong.

These same physicians do not invariably find it imperative to use such irrational methods in the treatment of diseases which manifest themselves in other organs of the body. Is it a misfortune that the anatomical structure and location are such as to render it possible that the uterus should be the one organ in the human body to suffer the maltreatment to which, in these modern days of gynecological wisdom, it is so often subjected?

The stomach, for instance, is, in common with other mucous lined organs, liable to local manifestations of disease. Is it to be regretted that in the emergency of hæmatemesis it is practically out of reach of the curette, or of the orthodox douche of a strong solution of persulphate of iron or of packing with carefully sterilized gauze?

All these scientific therapeutic agencies so strongly recommended in the treatment of uterine hemorrhage are lost to the poor suffering stomach, and life probably therefore jeopardized on account of its unfortunate location.

Seriously, is it not time to protest against such practice carried on under the name of Homœopathy, and against such teaching in our homœopathic schools? Why single out this special class of cases to be treated empirically, if we believe in Homœopathy and practice it in the treatment of diseases presenting other local manifestations? Do we not by such practice betray our disloyalty to Homœopathy and our lack of faith in its vital principles? If any of us believe in these practices, and deceive ourselves into thinking that we thereby radically *cure* disease, very well, but let us not call it Homœopathy. Later on, when by these suppressive measures the disease appears in another location and form, we have the battle to fight over again, and in all probability, at far greater disadvantage than if we had cured it at the outset, and certainly with increased danger to the patient.

These unscientific measures are as far as possible from Homœopathy; and, moreover, if we consider it necessary to practice empirically in one class of cases, does it not follow that we are liable, if not likely, to do it in all? And if so, where is our banner? Not over our heads, surely, but trailing in the dust. Our mistake is in lack of faith in our principles and lack of confidence in the power of our proved remedies. If we have failed in our results in the treatment of gynecological cases it is not the fault of our system of practice. It is from lack of discernment of the true and perfect picture of the case, from failure of selection of the proper remedy, or of insistence upon favorable hygienic conditions.

No surgeon overlooks or fails to emphasize the importance of certain conditions favorable to the union of a broken bone, or to the repair of a strained muscle or tendon. Let the gynecologist exercise equal wisdom and care in the treatment of what are called pelvic diseases. Let him explain especially the necessity of freedom from weight, stiffness, and pressure of the clothing upon the abdominal and pelvic organs, and then insist on the co-operation of the patient in this and every other necessary requirement.

This done, with the exercise of an ordinary degree of intelligence and common sense on the part of the patient, as to hygienic habits in general, the physician will find his carefully selected remedy doing most satisfactory and often most prompt and wonderful work, and that he has no necessity, or even excuse, for going outside of homœopathic therapeutics to cure his patient. Moreover, he will find that the gynecologist will become *the physician,* whose office—higher than that of any specialist, in the narrow sense in which that term is too apt to be used—is *to cure disease,* whether or not it may chance to exhibit prominent symptoms localized in the female pelvis, and therefore coming within the classification of diseases of women.

When once we come to the point of believing that all curable disease can be cured by Homœopathy, whatever its local manifestations may be and in whatever part of the human organism—and that this is the only immutable law of cure—then we shall be worthy to be called homœopathic physicians, for we shall *cure* our patients by following the law of similars.

Since it is not only possible but easy to do this, if only we are willing to work, why turn aside from this sure method to engage in that of making local applications of drugs not indicated in the case but used empirically in imitation of other practitioners who have no law of cure to guide them? In doing this it seems to me one shuts his eyes to several important facts.

*First.* A local manifestation or symptom of disease is not the disease itself, and the suppression of a symptom or group of symptoms is not the curing of the disease. If we wish to eradicate weeds from a lawn we do not hire a man to go over it picking off their blossoms. We insist on the importance of destroying the whole weed, root, branch, and blossom, and we accept nothing less than this as thorough work. How is it in the therapeutic field? Does a blossom of disease show itself in the form of leucorrhœa, of inflammation, or of ulceration? *Nip it off,* cries the popular voice, by the use of hot-water douches or the application of astringent or other drugs, conveyed to the supposed site of the disease by means of the medicated douche, the tampon, the tablet, or suppository. Is the offending organ out of place, from constitutional weakness or other cause? *Prop it up* with tampons, or a pessary. Is it a glandular enlargement or a morbid growth? *Cut it out.* Is it even dysmenorrhœa of perhaps a purely neurotic origin? *Remove the ovaries* and get rid of the pain.

No, no! This is not our legitimate work. These are not homœopathic methods. They suppress rather than cure the disease, and therefore, as avowed homœopaths, we ought to do better.

*Second.* Disease is not a material entity which has taken possession of the body through external media, and therefore to be forcibly ejected therefrom. This kind of warfare is not in harmony with the curing of disease "in the shortest, most reliable, and safest manner." We must remember that disease is a dynamic disturbance of the vital force, and that harmony must be restored by the dynamic action of a drug which has been properly prepared and administered—a drug which is capable of producing in the healthy organism a disease similar to the condition of the diseased organism.

Therefore the local symptoms which are found in this class of cases are of value to us, as in all other cases, as guides in our selection of the proper means of cure, and should not be suppressed by the indiscriminate and unscientific use of topical applications which are in no sense homœopathic to the case.

*Third.* We need to remember that it is not the direct action of the remedy which produces the cure, but the curative power of nature incited to reaction by the remedy. Nature is the physician. We are but her servants to discern and apply the subtile force whereby the "sweet bells jangled" shall be so re-attuned as to chime out again, in accordant notes, the matchless harmony of health.

And is there not something inspiring in the thought that to us is given this high privilege of service? To our master, Hahnemann, was given a yet higher privilege—that of discovering and formulating the law whereby such miracle may be wrought. To us, his humble followers, what honor can be higher than that of demonstrating this law in our daily work among the sick?

Is not this a kinship to recognize with pride and loyalty, and to honor by the constant exercise of the highest mental effort of which we are capable, to the end that we may faithfully and unswervingly apply this law to the healing of disease?

